# A novel approach to achieve semi-sustained drug delivery to the eye through asymmetric loading of soft contact lenses

**DOI:** 10.1016/j.heliyon.2023.e16916

**Published:** 2023-06-05

**Authors:** Malake Sarmout, Yutang Xiao, Xiao Hu, Aiym Rakhmetova, Leo H. Koole

**Affiliations:** Innovative Engineering Laboratory for Ocular Drug Delivery, Eye Hospital of Wenzhou Medical University, School of Ophthalmology and Optometry, Wenzhou, China

**Keywords:** Ocular drug delivery, Contact lens, Drug-eluting contact lens, Microparticles, Cornea, Rebamipide

## Abstract

Soft contact lenses are increasingly being explored as a vehicle for controlled delivery of ophthalmic drugs. However, traditional methods of drug-loading by soaking have limitations such as burst delivery and the release of drugs at the front side of the lens, leading to poor drug efficacy and systemic side effects. This study introduces a new methodology, termed asymmetric drug loading, whereby the ophthalmic drug ‘Rebamipide’ is attached to and released from the post-lens (=cornea-contacting) surface exclusively. The methodology involves using polymeric microparticles that carry a lipophilic crystalline ophthalmic drug at their surface. These drug-loaded microparticles first transfer the drug to the concave surface of the contact lens, and when worn, the drug is transferred again, now from the lens to the cornea. This is achieved through the diffusion of the drug from one hydrophobic microenvironment (the silicone moieties of the contact lens polymer network) to another hydrophobic microenvironment (the corneal epithelium) over a short pathway. The second drug transfer was observed and studied in experiments using an *ex vivo* porcine eye model. The results show that the drug amount that was absorbed by the cornea after applying the rebamipide-loaded contact lenses is approximately 3× (10.7 ± 3.1 μg) as much as the amount of rebamipide that gets transferred after the instillation of one eye drop (1% solution (*p < 0.001*). The new drug-loading method offers a practical and reproducible means of delivering ophthalmic drugs to the cornea through soft contact lenses. The drug payloads achieved are comparable to dosages used during eye drop therapy.

## Graphical abstract

Created with Biorender.com.

**Figure:**
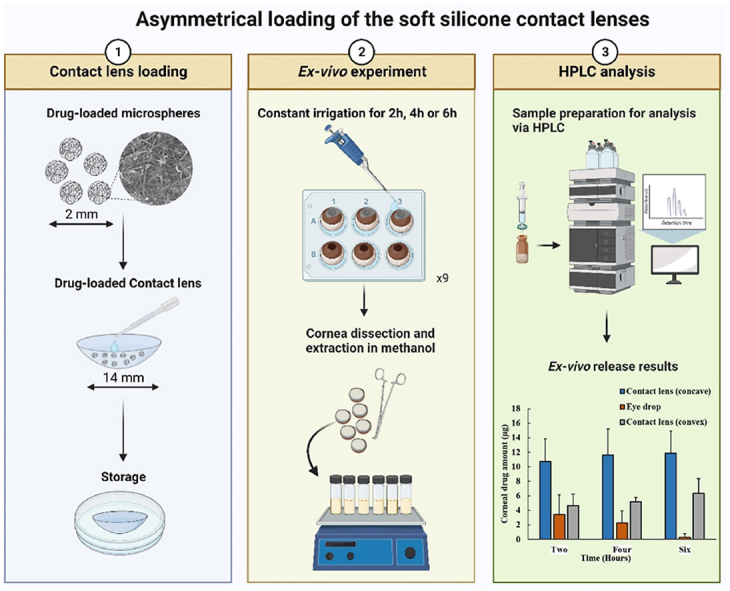
Schematic illustration of asymmetric loading of soft silicone contact lenses.

## 1 Introduction

Rebamipide is a successful ophthalmological drug used in the treatment of *keratoconjunctivitis sicca*, also known as dry eye syndrome (DES). The drug is a synthetic derivative of quinolone. It was originally developed in Japan, where it has been used to treat gastric ulcers and lesions associated with gastritis for over 30 years. Among rebamipide's most prominent activities are upregulation of the secretion of mucins and scavenging of oxygen free radicals [1,2]. In 2004, it was discovered that rebamipide could restore mucin concentrations in the cornea and conjunctiva of rabbits, in which mucin levels were first decreased by N-acetylcysteine [3,4]. Upregulation of mucins in the cornea and conjunctiva by rebamipide was also found in human DES patients, which led to the approval of the drug for the treatment of DES in Japan and the launch of rebamipide ophthalmic suspension in 2012 [5,6]. Since then, several clinical studies, mainly from Japan, have confirmed that rebamipide -administered as 1% or 2% eye drops- provides effective treatment for DES patients [[6], [7], [8], [9], [10], [11]]. A typical regimen is 4 drop installations daily for a period of 4 weeks.

The use of contact lenses (CLs) for drug delivery has emerged as a promising approach for the treatment of various ocular diseases. The eye is a complex and challenging organ for drug delivery due to various physiological barriers such as the corneal and conjunctival barriers, the tear film, and rapid clearance mechanisms [12]. Traditional topical ocular drug delivery methods such as eye drops or ointments often suffer from low bioavailability, poor drug absorption, and the need for frequent administration, leading to patient noncompliance and systemic side effects. In contrast, contact lenses are able to provide sustained drug release over an extended period of time, enabling improved drug efficacy. The literature provides a plethora of approaches by which drugs can be loaded onto/into CLs [[13], [14], [15], [16], [17], [18]], and numerous methods have been developed to steer the kinetics of in situ drug release to prevent burst release *in vivo* [[19], [20], [21], [22], [23], [24], [25]].

The mechanism by which drug-loaded CLs deliver their cargo to the cornea is diffusion, occurring mainly across the post-lens tear film, i.e., behind the lens [[26], [27], [28], [29]]. There, the flow of the tear fluid is slow; it has been estimated that the residence time is approximately 30 min which is far longer than normal tear flow and which enhances the drug's bioavailability. Drug molecules that leave the CL at the front side are removed relatively quickly with the tear flow and will not reach the cornea.

This has inspired us to search for a methodology to load CLs asymmetrically, i.e., to load the CL only on the hollow surface (the surface that will touch the cornea). Here, we describe such a loading technique. The CL used is ACUVUE® OASYS® with HYDRACLEAR® PLUS Technology, a product of Johnson & Johnson Vision, the drug that was loaded is rebamipide. The CL contains 38% water and consists of a silicone hydrogel biomaterial known as senofilcon A. The results show that The ACUVUE® OASYS® with HYDRACLEAR® PLUS soft contact lens loaded asymmetrically with rebamipide leads, after 2 h, to absorption of 10.7 ± 3.1 μg of the drug in the cornea, which is approximately 3× as much as the amount of rebamipide that gets transferred to the cornea after instillation of one eye drop (1% solution). These data reveal that the bioavailability of the drug is substantially improved upon using the new drug-loaded contact lens, as compared with eye drop therapy. The observations were made with the *ex vivo* porcine eye model, in which tear flow was mimicked.

Therefore, using the described method for asymmetric drug loading of soft contact lenses provides a novel treatment strategy for ophthalmologists. Asymmetric drug loading is achieved by micro-dosing the amount of drug loaded only on the cornea-facing side of the contact lens to meet the therapeutic needs, ensuring a consistent therapeutic drug concentration in the cornea. It will overcome the limitations of traditional methods, such as burst delivery, poor drug efficacy, and systemic side effects associated with eye drops. It is worth mentioning that this technique could be used for the controlled delivery of other ophthalmic drugs, making it a versatile tool in treating various ocular diseases. We report: (i), a new HPLC method for quantification of rebamipide; (ii), a practical procedure for asymmetric drug loading of CLs (iii), data on the drug cargo on these CLs, and experiments on the release of rebamipide in an *ex vivo* model (porcine eyes) in which the rebamipide-eluting CL is compared with rebamipide eye drops. Finally, we briefly discuss the limitations and possible pros and cons of this technique, as well as its versatility.

## 2 Materials and methods

### 2.1 Materials

Medical grade rebamipide (assay ≥98%) pure form was purchased from Hyper Chemicals Ltd (Hangzhou, China). HPLC-grade solvents (acetonitrile, methanol, ammonium acetate, and acetic acid) were purchased from Merck KGaA (Darmstadt, Germany). NaCl, KCl, CaCl_2_ and NaHCO_3_ were purchased from Aladdin Bio-Chem Technology company Ltd (Shanghai, China). Dulbecco's Modified Eagle Medium (DMEM), fetal bovine serum (FBS), penicillin and streptomycin were purchased from Sigma-Aldrich trading company Ltd (Shanghai, China), CCK-8 purchased from Dojindo Laboratories company Ltd (Kumamoto, Japan), ACUVUE® OASYS® with HYDRACLEAR® PLUS Technology contact lenses (38% water, 54% senofilcon A, diameter 14 mm, base arc 8.8 mm) were purchased from Johnson & Johnson Vision Care (Limerick, Ireland); Lot number L005HNN840; Production date 2022-06-04. Methyl methacrylate (MMA), hydroxyethyl methacrylate (HEMA), ethylene glycol dimethacrylate (EGDMA), poly(vinylalcohol) (PVA), [(CH_2_–CHOH)_n_, 98–99% hydrolyzed], poly(vinylpyrrolidone) (PVP), [(C_6_H_9_NO)_n_, BASF K-30], poly(ethylene glycol) (PEG), [HO(CH_2_CH_2_O)_n_H; Mn = 1000] and *tert*-butyl 2-ethylhexaneperoxoate (initiator) were purchased from Macklin Biochemical Co., Ltd. (Shanghai, China); 4-Iodobenzoyl-oxo-ethyl methacrylate (4-IEMA) was prepared as described previously [30], MMA was distilled at atmospheric pressure and stored at −20 °C, and the other chemicals were used as received. Freshly excised porcine eyes were obtained from a local abattoir. Deionized ultra-pure water was obtained using the Milli-Q ® system Merck Millipore (France). Simulated tear fluid (STF) was prepared by dissolving 6.7 g NaCl, 2.2 g NaHCO_3_, 1.4 g KCl and, 0.1 g CaCl_2_ in 1 L of distilled deionized ultra-pure water [31]. Ammonium acetate buffer was prepared by dissolving 1.54 g of ammonium acetate (0.02 mol) in 1 L of deionized ultra-pure water; pH was adjusted to 4.4 by carefully adding acetic acid. All other reagents and solvents used in this study were of analytical grade.

### 2.2 Chromatographic analysis

We developed a method for HPLC analysis of rebamipide. All analyses were performed with Agilent 1260 Infinity Ⅱ HPLC system Agilent Technologies (Waldbronn, Germany) equipped with ChemStation software, G7111A 1260 Quaternary Pump VL, G7114A 1260 VWD detector, G7129A 1260 vial sampler and Poroshell 120 EC- C18 (4.6 mm I.D. × 150 mm, 4 μm) column Agilent Technologies (St. Louis, Missouri, USA), UV detection was used (λ = 250 nm). All samples were filtered through a 0.1 μm Biofil syringe filter membrane prior to injection. Optimization was performed in several steps outlined in the supplementary materials to meet the international standards [32].

#### 2.2.1 Analysis

The optimal parameters for the HPLC analysis were as follows: Column temperature 35 °C. Mobile phase: ammonium acetate buffer (pH 4.4), acetonitrile, and methanol (55:30:15); isocratic separation. Flow rate: 1.0 mL/min. Detection: UV at 250 nm. Injection volume 6 μL. A calibration curve was constructed by preparing a stock solution of rebamipide (20.0 mg) in methanol (100 mL). The mixture was sonicated for 30 min (water bath regulated at 45 °C) until no drug residue was observed. Standard solutions were prepared by diluting the stock solution in methanol to the following selected concentrations 1, 5, 10, 15, 25, 50, 75, and 100 μg/mL. Before injection into the HPLC, samples were filtered through a syringe filter 0.1 μm pore-size membrane. Next, three injection volumes of each concentration were auto-injected. The calibration curves and the regression equations were derived by plotting the peak area ratios against the corresponding concentration of each sample (μg/mL) (supplementary material S3).

### 2.3 Chemical synthesis of the polymeric microparticles

Firstly, a detergent solution was prepared in a 1 L beaker by dissolving PVA (12.8 g), PVP (5.8 g) and PEG (9.72 g) in 400 mL of water under mechanical stirring during 24 h at ambient temperature until the PVA completely dissolved. Next, the mixture was transferred into a 1-L round bottom flask. The flask was immersed in an oil bath and placed on a magnetic stirrer equipped with a temperature control system. The flask's content was stirred (300 rpm) and heated to 90 °C. Then, the monomer mixture consisting of MMA (2.00 g, 20.0 mmol), HEMA (0.26 g, 2.0 mmol), EGDMA (1.98 g, 10.0 mmol), 4-IEMA (1.89 g, 5.0 mmol) and initiator (1.05 g, 4.0 mmol)] was added dropwise via a polypropylene pipette and the temperature with stirring continued for 60 min. After that, the heating was turned off, cold water (200 mL) was added, and after the mixture cooled to room temperature, stirring was stopped. Particles were worked up through decantation; they were washed with water repeatedly (>6 times), washed with ethanol 96% (once), and washed with water again (3 times). Clean particles (microspheres) were collected on a Petri dish and dried in the oven overnight at 37 °C. The microspheres consist of a poly(methacrylate)-type three-dimensional network and have passed extensive testing for biostability and biocompatibility in the past [[33], [34], [35], [36], [37]].

### 2.4 In vitro cytotoxicity

The Human corneal epithelial cells (HCECs) were cultured in DMEM supplemented with 10% FBS, penicillin (100 units/mL), and streptomycin (100 μg/mL). All cultures were maintained at 37 °C under 5% CO_2_ for 24 h. To evaluate the cytotoxicity of the microspheres and the effect of the diameter (μm) on the cell viability, cells were seeded in a 24-well plate divided into four groups (Control = no microparticles, 100–200 μm, 200–300 μm, and 300–500 μm) at an initial density of 20,000 cells/well and incubated for 24 h. Subsequently, the medium was replaced with fresh DMEM, and the cells were incubated with 1 mg of different diameters of microspheres for 4 h. Then, the cells were rinsed with PBS buffer, and a fresh culture medium was added. After 24 h of incubation, 400 μL fresh culture medium with 40 μL CCK-8 solution were added into each well, followed by incubating at 37 °C for another 4 h. Finally, the absorbance was measured at 450 nm by a microplate reader (SpectraMax i3x, Molecular Devices), and the cell viability was determined according to the following equation:

$$Cell\;viability\;\left(\%\right)=\frac{A_{450\;nm,sample}-A_{450\;nm,blank}}{A_{450\;nm,control}-A_{450\;nm,blank}}\times 100\%$$

where A_450 nm, sample_ and A_450 nm, control_ are the absorbance in the presence and absence of microparticles, respectively. A_450__nm_, _blank_ is the absorbance of the well with 40 μL of CCK-8 without cells. All the samples were evaluated in n = 6.

### 2.5 Loading of microparticles with rebamipide

Firstly, a drug solution was prepared in a glass vial by dissolving rebamipide (150 mg) in DMSO (2.00 mL). Then, 500 mg of microparticles (diameter range 300–500 μm) were carefully added. The mixture was placed on a shaker at low speed (75 rpm) at room temperature for 24 h. Next day, the supernatant was removed as much as possible using a syringe with a thin needle, and the particles were spread at the bottom of the vial then placed horizontally in a refrigerator (4 °C) for 1 h. Next, 1.00 mL of cold water (4 °C) was added, and the vial was gently shaken for 30 s and left to stand at room temperature for 2 h. Later, the particles were washed extensively with ultra-pure water (>8 times), collected on a Petri dish, and allowed to dry overnight in the oven at 37 °C. The dry particles were transferred to the sieve with an aperture of 300 μm i.e., the sieve from which they were collected originally. This treatment (sieving machine, 5 min) represents a mechanical challenge to the drug-loaded particles; loosely bound drug crystals detached and passed the sieve, thus leaving stable drug-loaded microparticles only.

### 2.6 Evaluation of drug loading of microparticles and contact lens

An aliquot (10.0 mg) of the drug-loaded particles weighed (M) was incubated with methanol (2.00 mL) and gently agitated for 24 h. The concentration of rebamipide in the supernatant (mp) was determined by HPLC, and the loading capacity was calculated according to the following equation:

Loadingofmicrospheres(%)=100×mp/M

### 2.7 Asymmetric loading of commercial contact lens

Firstly, the contact lenses (CLs) were taken from their original package and placed in a Petri dish (with the concave (hollow) side upward, Fig. 5B) pre-prepared with a moist filter paper by adding 200 μL of ultrapure water. Next, a pre-weighed amount of the loaded microparticles (7.0 mg, 10.0 mg, or 12.0 mg) was carefully added into the middle of the contact lens, followed by 10.0 μL of ethanol. The microparticles were carefully spread to form a near-circular distribution (diameter ∼ 6 mm), and to decrease the physical interaction between the microspheres. Petri dishes were sealed and placed in the fridge. Another 10.0 μL of ethanol was added to each sample after 6 h of incubation. The following day, each contact lens was washed with the contact lens solution to remove the particles, then placed in a new Petri dish immediately or stored in the freezer at −18 °C. To investigate the amount of drug loaded onto the contact lens, the latter was placed in a tube with 2.00 mL of HPLC grade methanol, then transferred onto an incubator shaker for 24 h. The next day, each sample was filtered and transferred to auto-sampler vials for HPLC analysis according to the above-mentioned method.

### 2.8 Drug delivery to porcine eyes

Porcine eyes were collected on the day of slaughter from a local abattoir, transported at 4 °C, and used for experimentation within 6 h. Before the experiment, the eyes were carefully checked for damage or scarring, and any excess fat was eliminated using scissors. Then, the eyes were placed with the cornea facing up in individual wells of a 6-well plate (×6) containing 1 mL PBS to keep them hydrated and placed in a 35 ± 1 °C water bath for 10 min. After that, rebamipide-loaded contact lenses on the concave “correct” side (groups 1–3, see below) or 43 μL of Mucosta® (1%; groups 4–6, see below) or rebamipide-loaded contact lenses on the convex “wrong” side (groups 7–9, see below) were applied (Table 4). To simulate steady tear flow, freshly prepared STF was instilled into each eye at a rate of 2.4 mL/h (by dripping 200 μL every 5 min). Finally, the eyes were kept irrigated in the water bath for 2 h (groups 1, 4 and 7), 4 h (groups 2, 5 and 8), or 6 h (groups 3, 6 and 9).

After each experimental time interval, the eyes were removed from the water bath and the contact lenses removed. Using surgical scissors and forceps, each cornea was dissected and then extracted twice in methanol. Each cornea was placed in a 5 mL glass vial with 1.00 mL of HPLC grade methanol and, put on a Bluepard shaking incubator (220 rpm) at room temperature overnight. The Next day, each cornea was removed and placed in a second vial with 1.00 mL of fresh HPLC grade methanol; the previous methanol fraction was preserved at 4 °C. The vials containing corneas were then put back into the shaking incubator again overnight. The next day, the two methanol fractions were combined in one vial, filtered with a 0.22 μm Biofil filter membrane and evaporated overnight in a 50 °C oven [31,38]. The resulting dry residue was reconstituted with 1.00 mL fresh HPLC-grade methanol and transferred to auto-sampler vials for HPLC analysis according to the abovementioned method.

#### 2.8.1 Scanning electron microscopy (SEM)

SEM and energy-dispersive X-ray (EDX) images were recorded using a Hitachi SU 8010 instrument (Tokyo, Japan) at an acceleration voltage of 5 kV and a working distance of 2 mm and 12 mm for particles and drug-loaded contact lens respectively. Particles were mounted on a stainless-steel stub using double-sided tape, while the drug-loaded contact lens was mounted on the steel (stub) and then left at room temperature to air dry for 48 h completely. Both of the samples were metalized with Pt using a Leica EM ACE600 sputter coater. The thickness of the Pt layer was approximately 5 nm (sputter time 200 s).

## 3 Results

### 3.1 Preparation of the microspheres, in vitro cytotoxicity tests with HCECs

**Fig. 1:**
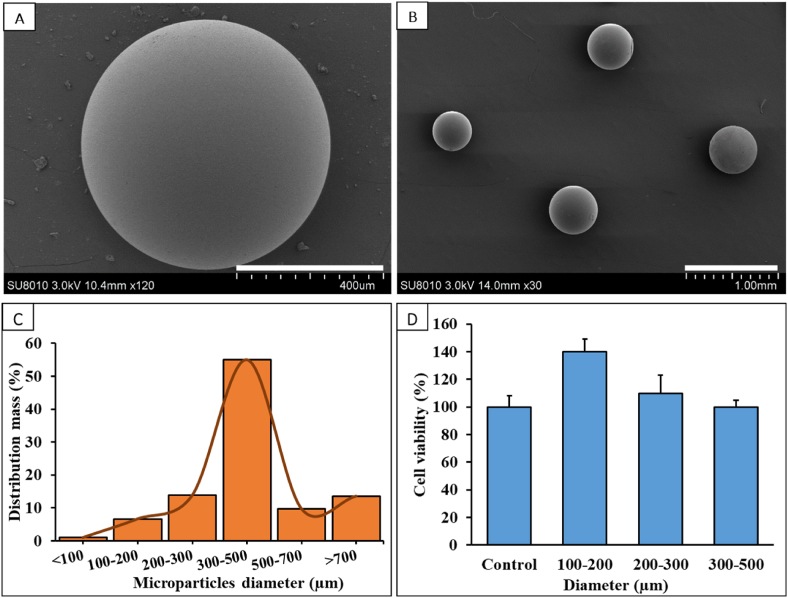
SEM images, diameter distribution and cytotoxicity analysis of the polymeric microspheres studied in this work. (A), Close-up view of a typical microsphere, diameter approximately 600 μm, note the smooth surface. Scale bar = 400 μm. (B), Four typical microspheres with diameter in the range 300–500 μm. Scale bar = 1000 μm. (C), Diameter distribution (expressed in mass %) of microparticles from a typical synthesis run. Diameter ranges from <100 to >700 μm. (D), Results from CCK8 in vitro cytotoxicity tests.

Synthesis of the microparticles proceeded smoothly. Due to the statistical nature of stirred suspension polymerizations [39] a distribution of differently-sized microspheres is obtained in each synthesis run. Size sorting was achieved through sieving. Fig. 1A and B shows representative SEM images of the synthesized microspheres. Fig. 1C compiles the size distribution from a typical synthesis run. Using the well-known CCK8 cytotoxicity test, the microspheres were incubated with cultured HCECs, and cell viability was measured. The results are shown in Fig. 1D.

### 3.2 Analysis of rebamipide by HPLC

A method for HPLC analysis was developed, as we could not find a suitable procedure in the literature. Our optimization led to the parameters as compiled in Table 1.

**Table 1:** Optimized parameters of HPLC and results from LOD and LOQ analysis.

Parameter		Parameter	
Column	Poroshell 120 EC- C18	Intercept (a)	−2.504
Column temperature	35 °C	Slope (b)	16.73
Flow rate	1.0 mL/min	Correlation coefficient (R^2^)	0.9999
Separation mode	Isocratic	Standard deviation of residuals (Sy/x)	6.34
Mobile phase	Ammonium acetate buffer/ACN/MeOH	Standard deviation of intercept (Sa)	4.59
Detection	UV	Standard deviation of slope (Sb)	0.082
Detection wavelength	λ = 250 nm	Limit of detection (LOD) (μg mL^−1^)	0.07
Linearity range (μg mL^−1^)	1–100	Limit of quantitation (LOQ) (μg mL^−1^)	0.5

### 3.3 Loading of the microparticles with rebamipide

**Fig. 2:**
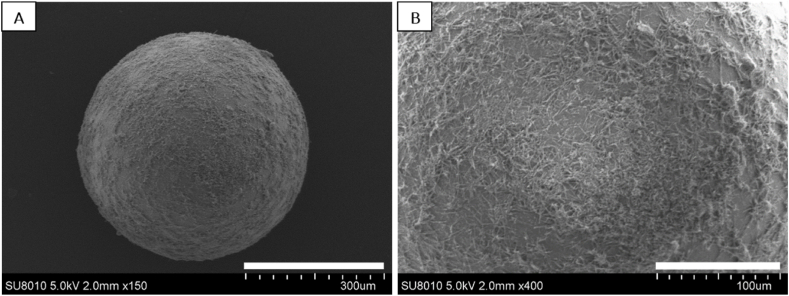
Drug-loaded microparticles. (A), SEM of a rebamipide-loaded microsphere. Scale bar = 300 μm; (B), Magnification of image (A) showing the crystalline drug on the microsphere's surface. Scale bar = 100 μm.

We selected microspheres in the diameter range 300–500 μm for drug loading. These particles are mid-size (Fig. 1A) and large enough to be manipulated, e.g, with tweezers. Aliquots (n = 8) of drug-loaded particles (10.0 mg each) were taken, transferred to a 10 mL glass vial, and incubated with methanol (2.0 mL). Concentrations of the drug were then measured (HPLC), and the cargo on particles was calculated. The results are compiled in Table 2. It followed that the drug loading was 37.9 ± 1.0 μg rebamipide per mg of particles. Alternatively stated, the loading of the particles was 3.8% on average. Fig. 2A shows some representative SEM images of the rebamipide-loaded microspheres; note the adhered needle-shaped drug crystals at the periphery of the particles (Fig. 2B).

**Table 2:** Results from HPLC analyses of microspheres loaded with rebamipide. Note the reproducibility.

Aliquot #; each 10.0 mg	Amount of drug immobilized on the particles (μg)	Loading percentage (%)
1	366.6	3.7
2	363.2	3.6
3	381.6	3.8
4	395.1	4.0
5	379.5	3.8
6	388.9	3.9
7	377.1	3.8
8	376.3	3.8
Mean ± SD	379 ± 10	3.8 ± 0.1

### 3.4 Transfer of rebamipide from the particles to the contact lens

Three groups of contact lenses (n = 8 in each group) were incubated with drug-loaded microparticles (see Materials & methods). The groups were charged with either 7, 10 or 12 mg of the particles.

#### 3.4.1 Analysis by HPLC

After incubation, removal of the particles and washing, the drug was extracted (methanol) and analyzed by HPLC. The resulting loadings of the contact lenses are compiled in Table 3.

**Table 3:** Results of HPLC analysis of rebamipide-loaded contact lenses. First, second and third group of CLs were incubated with 7, 10 or 12 mg of the drug-loaded microspheres, respectively, see text, p < 0.001.

	Group 1	Group 2	Group 3
Mass of drug-loaded microparticles during incubation (mg)	7	10	12
Lens #	Amount of drug transferred to the contact lens (μg)		
1	32.089	49.226	39.464
2	29.724	44.699	38.662
3	33.584	50.393	38.799
4	26.349	44.829	37.36
5	29.477	48.113	39.893
6	31.259	46.072	37.519
7	29.571	45.521	38.943
8	30.139	47.673	36.576
Mean ± SD	30.3 ± 2.0 μg	47.1 ± 2.0 μg	38.4 ± 1.1 μg
% drug transferred from the micro-particles to the CL	11.4	12.4	10.1

**Table 4:** Results of the analyses of rebamipide that has been absorbed by porcine corneas after delivery from the loaded contact on the concave side lenses (groups 1–3), after instilling one rebamipide eye drop (Mucosta®, 1%, groups 4–6) or from the loaded contact on the convex side lenses (groups 7–9).

Group	Contact time (h)	Drug transferred from the concave side of the contact lens to cornea (μg)							Mean ± S.D. (μg)	Percentage of the amount of drug administered ± SD
1	2 h	11.6	14.7		7.5	12.6	6.5	11.6	10.7 ± 3.1	22.7 ± 6.6
2	4 h	11.3	11.2		16.7	12.0	13.1	5.7	11.6 ± 3.6	24.6 ± 7.5
3	6 h	9.2	14.4		14.8	8.1	14.5	10.2	11.9 ± 3.1	25.2 ± 6.5
Rebamipide eye drops (43 μL, 1%; Mucosta®)										
4	2 h	1.1		7.4	4.5	4.7	<LOD	2.8	3.4 ± 2.7	0.8 ± 0.6
5	4 h	4.6		<LOD	2.8	1.5	3.5	1.3	2.3 ± 1.7	0.5 ± 0.4
6	6 h	3.2		<LOD	0.5	1	<LOD	<LOD	0.3 ± 0.5	0.1 ± 0.1
Drug transferred from the convex side of the contact lens to cornea (μg)										
7	2 h	4.1		6.2	5.3	6	4.5	2	4.7 ± 1.6	10.0 ± 3.4
8	4 h	4.6		5.8	5.7	5.64	4.8	4.5	5.2 ± 0.6	11.1 ± 1.3
9	6 h	7.2		2.3	7.1	6.7	7.3	7.5	6.3 ± 2.0	13.4 ± 4.3

The percentages of drug transfer were calculated as follows:

$$\%\;drug\;transferred=\frac{Mean\;mass\;of\;drug\;measured\;on\;the\;contact\;lens}{Calculated\;mass\;of\;drug\;present\;on\;the\;microparticles}\times 100$$

It appears that the efficacy of the drug transfer is 10–12% in each case. It is important to compare the drug cargo of the contact lenses with the amount of rebamipide that is normally instilled in the eye during eye drop therapy. Dispersions containing either 1 or 2% rebamipide are normally used in eye drop formulations [6]. Hence, the concentration in the eye drops is 10 mg/mL (1%) or 20 mg/mL (2%). Per eye drop (approximately 30 μL) [40], this is 10,000 * 30/1000 = 300 μg (1%) or 600 μg (2%). It follows that the amount of rebamipide that is introduced into the eye using our loaded contact lenses is substantially lower than in the case of eye drop instillation, namely (30–47 μg for the loaded contact lens (Table 4, Graph 2) versus 300 (1%) or 600 μg (2%) per eye drop. However, taking into account that 90–95% of eye-drop-instilled drugs are lost due to blinking and lachrymal drainage [3], the comparison becomes favorable for the drug-loaded contact lens: 30–47 μg versus 15–30 μg for the 1% eye drops and 30–60 μg for the 2% eye drops.

#### 3.4.2 Analysis by SEM and SEM-EDX

**Fig. 3:**
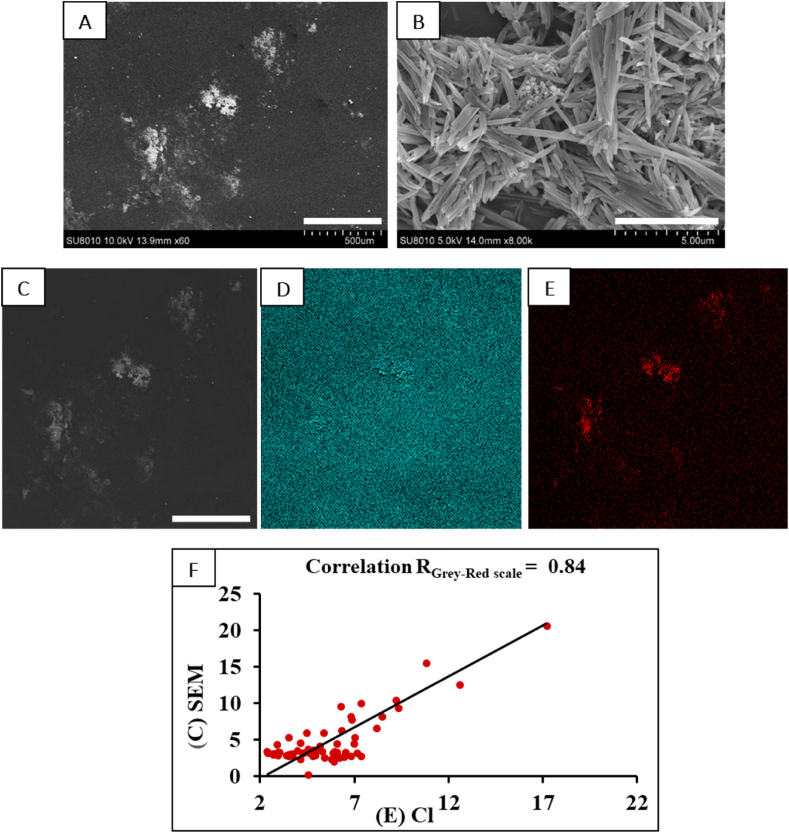
Characterization of a drug (rebamipide)-loaded contact lens. (A), SEM image of the concave surface of a rebamipide-loaded CL. The light-grey regions indicate the presence of the drug; these spots correspond with the location of the drug-carrying microspheres during the drug-loading procedure. Scale bar = 500 μm. (B), Close-up SEM image of one of the light-grey spots in A. Note the presence of nano-sized needle-shaped rebamipide crystals, adhering to the lens' surface. (C), Reference SEM image of the same area of the concave surface of the CL, as seen in (A). (D) and (E) SEM-EDX images revealing the presence of the elements carbon (green, in D) and chlorine (red, in E). Note that carbon is present in both the CL's polymer structure and rebamipide, i.e., green color is seen anywhere in (D). Yet, elevated carbon concentrations can be discerned in the regions corresponding with the light grey regions in (C). Note furthermore that chlorine is part of the drug but not of the CL. Hence, (E) merely shows red regions in a black (chlorine-free) background. Note the correspondence of the red regions in (E) and the light-grey regions in C, proving that the light-grey regions in (C) and (A) are due to rebamipide. Scale bar in C = 500 μm; this also applies to (D) and (E). (F), quantification of the correlation of the SEM-EDX plots (C) and (E). (For interpretation of the references to color in this figure legend, the reader is referred to the Web version of this article.)

The drug loaded-microparticles and drug-loaded contact lenses were further studied with SEM. The spherical shape of the microspheres covered with microneedles of rebamipide is shown in Fig. 2A. A magnification of ×100 clearly shows the crystalline structure of the drug attached to the surface of the microsphere (Fig. 2B). The hollow surface of the loaded lenses was scanned using energy-dispersive X-ray (SEM-EDX) analysis. Representative SEM images and SEX-EDX elemental maps are shown in Fig. 3. For SEM-EDX, it was very helpful that rebamipide contains chlorine, whereas the contact lens does not. There is a clear correlation between the grey SEM image (Fig. 3C) and the red (chlorine, SEM-EDX) image in Fig. 3E. The correlation was substantiated further after quantitative image analysis (Image J, Fig. 3F). It can be concluded that both SEM and SEM-EDX provided clear evidence for the presence of rebamipide on the inner surface of the loaded contact lens (Fig. 3A and B). It must be mentioned that inspection of the other surface of the contact lens did not show the presence of rebamipide there, i.e., our procedure leads to asymmetric loading of the lens, as anticipated.

### 3.5 Transfer of rebamipide from loaded-contact lens to the cornea and comparison with eye drops (Mucosta®, 1%)

**Fig. 4:**
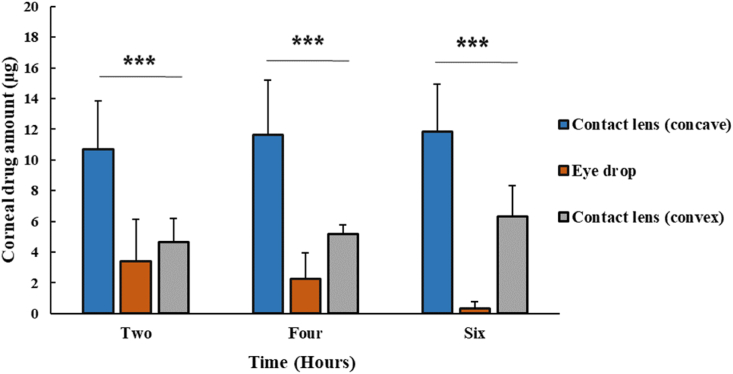
*Ex-vivo* porcine eyes experiment results. Quantities of rebamipide delivered to the cornea (μg) by means of rebamipide-loaded contact lenses on the concave or convex side and 1% concentration eye drop after 2 h, 4 h, and 6 h (n = 6 ± SD), *** = *p < 0.001*.

Experimental data were obtained with 6 groups of 6 porcine eyes while tear flow was mimicked. The amounts of drug transferred from the CLs to the corneas were analyzed by HPLC, using excised corneas. Contact times were 2, 4 or 6 h. Control experiments using rebamipide eye drops were included. The results are compiled in Table 4. After 2 h, 10.7 ± 3.7 μg (n = 6) of rebamipide was transferred to the cornea, and this amount increased only marginally after 4 h (11.6 ± 3.6 μg, n = 6) or 6 h (11.9 ± 3.1 μg, n = 6). If compared to the amount of drug present on the CLs, the amounts transferred were in the range 20–25%. The control experiments, using 43 μL of 1% eye drop formulation, delivered 3.4 ± 2.7 μg (n = 6) to the cornea after 2 h. After 4 and 6 h, this amount was decreased to 2.3 ± 1.7 μg (n = 6) and 0.3 ± 0.5 μg (n = 6), respectively. All data are collected in Fig. 4. Clearly, the amount of drug delivered via the drug-loaded CLs is much higher in comparison with the eye drop route. A second set of control experiments was done to further challenge the idea of asymmetric drug delivery. Here, each drug-loaded CL was reversed prior to placement on the cornea. The drug-loaded surface now faced upward (convex, toward the air) rather than downward (concave). *A priori*, this arrangement was expected to reduce the amount of drug transferred to the cornea. The results of the 2nd control series are also provided in Table 4 and Fig. 4. Remarkably, the reversed placement of the CLs also leads to delivery of the drug to the corneas, albeit less than for the “correct” arrangement. After 2 h, 4.7 ± 1.6 μg (n = 6) rebamipide was transferred, and after 2 and 4 h this amount increased to 5.2 ± 0.6 μg (n = 6) and 6.3 ± 2.0 μg (n = 6) respectively. This shows that asymmetric drug delivery occurs indeed, but not in an absolute sense. Most likely, the explanation is that the drug can penetrate the contact lens (by diffusion) prior to and during release to the cornea.

**Fig. 5:**
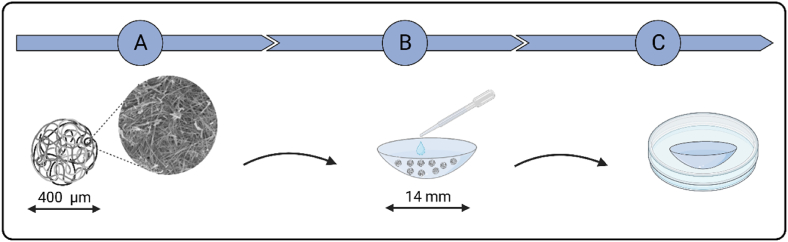
Schematic illustration of the method for asymmetric drug loading of soft silicone CLs, as developed and followed in this study. (A) Drug-loaded microspheres, diameter range 300–500 μm; (B) Assembly of the drug-loaded microspheres (typically 10 mg) in the center of the concave side of the CL. Application of ethanol (10 μL), see text; (C) particles removed by washing with CL fluid, the drug-loaded lens is stored in its original case.

## 4 Discussion

This study shows that CLs can be loaded with an ophthalmic drug on their concave (= hollow) side, by applying polymer microparticles which have the drug adhered physically to their surface. When such a CL is worn, its drug-loaded surface (sometimes also called “post-lens surface”) lies adjacent to the cornea, which provides a short diffusion pathway in an environment where the tear flow is slowed down [41,42]. These are conditions that favor drug release to the eye. The loading method is schematically depicted in Fig. 5.

The first step is the chemical synthesis of the polymer particles (microspheres). These are manufactured in a process that is known as suspension polymerization. This chemical reaction starts with the formation of discrete oil-like droplets consisting of initiator, methacrylic monomers, and crosslinkers within a stirred aqueous continuous phase with dissolved surfactant and/or polymeric stabilizers. The polymerization reaction occurs within each of the monomer droplets, which implies that polymeric microspheres are obtained as a statistical distribution of diameters (Fig. 1C) [43].

The second step involves creating and adhering small drug crystals at the surface of the polymer microparticles. This is achieved through incubation of the polymer microspheres in a solution of the drug in DMSO, followed by abrupt crystallization of the drug-induced by a sudden change of the medium from DMSO (solvent for the drug) to water (non-solvent for the drug) [36]. Analysis of the drug-loaded microparticles by HPLC has shown that the loading percentage of the microparticles is 3.8 ± 0.1%. The data (Table 3) show that the reproducibility (n = 8; SD < 3%) of our technique to load microparticles is very good.

In the third step, drug-loaded microparticles (e.g., 7, 10, or 12 mg) are carefully placed adjacently and centrally on the concave side of the wet CL (Fig. 5).

In the fourth step, the particles are removed carefully, and the CL is washed with contact lens fluid.

It is of interest to examine the drug loading on the contact lenses somewhat closer and to draw a comparison with the application of rebamipide eye drops, for which two formulations (1% and 2%) are in clinical use [[6], [7], [8], [9], [10], [11]]. Assuming that one eye drop corresponds with a volume of 30 μl [40], it appears that an eye drop will instill 300 μg (1%) of rebamipide in the eye. Assuming that 90–95% of each drop gets spilled due to blinking and lachrymal drainage [44,45], it follows that 15–30 μg (1%) of the drug is “bioavailable”. In our opinion, this compares well with the 47 ± 2.0 μg, found for the rebamipide-loaded CLs of group 2 in Tables 4 and in light of the great uncertainty regarding the amount of drug that effectively reaches the eye after the instillation of an eye drop [46], we also believe that the variations encountered in the drug loading of the CLs (Table 4) are acceptable.

The most important results of this study are listed in Table 4, where the drug transfer to the cornea (in the *ex vivo* porcine eye model) is characterized. Three series of experiments were done: one in which is the drug is delivered from the concave (hollow) side of the contact lens (“correct”), one in which the drug is delivered by eye drops (1st control), and one in which the drug is delivered from the convex side of the CL (air-facing side, “wrong”; 2nd control). These data reveal several points: (i) drug delivery from the “correctly” placed CL is much more effective than drug delivery from eye drops. After 2 h, for example, the contact lens delivered 10.7 ± 3.1 μg of the drug to the cornea versus 3.4 ± 2.7 μg for the eye drop route. (ii) Reversing the drug-loaded CLs has a clear effect on delivery to the cornea; in this case, 4.7 ± 1.6 μg of the drug was transferred to the cornea after 2 h. In other words, this is approximately half the amount corresponding to “correct” placement and slightly more than the amount delivered by eye drops. The difference between correct and wrong placement reflects the asymmetry of the drug-loaded contact lenses. Interestingly, the asymmetry is not absolute, which may indicate that diffusion of the drug across the contact lens is occurring.

It is of interest to note that the corneal epithelium is known to be markedly hydrophobic [[46], [47], [48]]. Lipophilic ophthalmic prodrugs such as ester prostaglandin analogs have a tendency to diffuse into the corneal epithelium. The latter is most relevant to the mechanism of action of our CLs carrying lipophilic rebamipide. The presence of the hydrophobic epithelium adjacent to the rebamipide-loaded surface of the CL likely facilitates the transfer of the drug to the corneal epithelium, i.e., from one hydrophobic microenvironment to another hydrophobic microenvironment.

It is important to bear in mind that the *ex vivo* porcine model has substantial shortcomings. Its significance is limited since drug release proceeds merely under static conditions. The living eye provides a dynamic environment for tear fluids and CLs; multiple movements affect drug release. It is known that blinking is particularly dominant; Every blink causes slight changes in the shape of the contact lens, which in turn can pump portions of the post-lens tear film in and out [49,50]. For this and other reasons, further experiments with animal models *in vivo* are currently ongoing in this laboratory.

## 5 Conclusion

This study discloses a new technique for drug-loading of soft contact lenses which are intended for controlled delivery of ophthalmic drugs to the cornea. The loading method is practical and yields reproducible drug payloads comparable to effective dosages used during eye drop therapy. An *ex vivo* porcine eye model, including simulated tear flow, reveals that improved drug bioavailability can be achieved, as compared to the instillation of an eye drop. Asymmetric drug loading of contact lenses holds promise for several reasons: (i), it can help to minimize the spilling of ophthalmic drugs and hence help to prevent unwanted systemic side effects of ophthalmic medication; (ii) *in vivo* drug release involves very short diffusion pathways within the post-lens space. This is expected to be much more effective in comparison with drug-releasing contact lenses, which were loaded through drug-soaking; (iii), the method only brings well-known and generally trusted biomaterials (i.e., the soft silicone contact lens and the drug) into contact with the cornea.

## Author contribution statement

Malake Sarmout: Conceived and designed the experiments; Performed the experiments; Analyzed and interpreted the data; Wrote the paper.

Xiao Yutang: Performed the experiments; Analyzed and interpreted the data.

Hu Xiao: Performed the experiments; Analyzed and interpreted the data; Contributed reagents, materials, analysis tools or data.

Leo H. Koole: Conceived and designed the experiments; Analyzed and interpreted the data; Contributed reagents, materials, analysis tools or data; Wrote the paper.

## Data availability statement

Data included in article/supplementary material/referenced in article.

## Funding

This study was financed through personal grants from 10.13039/100007835Wenzhou Medical University and Zhejiang Province.

## Declaration of competing interest

The authors declare that there are no conflicts of interest. The sponsors had no role in the design, execution, interpretation, or writing of the study.

## References

[1] Zvyaglova M.Yu, Knyazev O.V., Parfenov A.I. (2020). Pharmacological and clinical feature of rebamipide: new therapeutic targets. Ter. Arkh..

[2] Hori Y. (2018). Secreted mucins on the ocular surface. Invest. Opthalmol. Vis. Sci..

[3] Eghtedari Y., Oh L.J., Girolamo N.D., Watson S.L. (2022). The role of topical N-acetylcysteine in Ocular therapeutics. Surv. Ophthalmol..

[4] Fukuda M., Takeda N., Ishida H., Seki Y., Shibata N., Takahashi N. (2022). Benzalkonium chloride-induced corneal epithelial injury in rabbit reduced by rebamipide. J. Ocul. Pharmacol. Therapeut..

[5] Kashima T., Akiyama H., Kishi S., Itakura H. (2014). Rebamipide ophthalmic suspension for the treatment of dry eye syndrome: a critical appraisal. Clin. Ophthalmol..

[6] Kinoshita S., Awamura S., Oshiden K., Nakamichi N., Suzuki H., Yokoi N. (2012). Rebamipide (OPC-12759) in the treatment of Dry Eye: a randomized, double-masked, multicenter, placebo-controlled phase II study. Ophthalmology.

[7] Ueda K., Matsumiya W., Otsuka K., Maeda Y., Nagai T., Nakamura M. (2015). Effectiveness and relevant factors of 2 % rebamipide ophthalmic suspension treatment in Dry Eye. BMC Ophthalmol..

[8] Shrivastava S., Patkar P., Ramakrishnan R., Kanhere M., Riaz Z. (2018). Efficacy of rebamipide 2% ophthalmic solution in the treatment of Dry eyes. Oman J. Ophthalmol..

[9] Kobashi H., Kamiya K., Shimizu K. (2017). Randomized comparison between Rebamipide ophthalmic suspension and Diquafosol ophthalmic solution for Dry Eye after penetrating keratoplasty. J. Ocul. Pharmacol. Therapeut..

[10] Koh S., Inoue Y., Sugmimoto T., Maeda N., Nishida K. (2013). Effect of rebamipide ophthalmic suspension on optical quality in the short break-up time type of Dry Eye. Cornea.

[11] Sakane Y., Yamaguchi M., Shiraishi A. (2019). Retrospective observational study on Rebamipide ophthalmic suspension on quality of life of dry eye disease patients. J. Ophthalmol..

[12] González-Chomón C., Concheiro A., Alvarez-Lorenzo C. (2013). Soft contact lenses for controlled ocular delivery: 50 years in the making. Ther. Deliv..

[13] Shikamura Y., Yamazaki Y., Matsunaga T., Sato T., Ohtori A., Tojo K. (2015). Hydrogel Ring for topical drug delivery to the ocular posterior segment. Curr. Eye Res..

[14] Liu D.E., Dursch T.J., Taylor N.O., Chan S.Y., Bregante D.T., Radke C.J. (2016). Diffusion of water-soluble sorptive drugs in Hema/MAA Hydrogels. J. Contr. Release.

[15] Zhu Q., Liu C., Sun Z., Zhang X., Liang N., Mao S. (2018). Inner layer-embedded contact lenses for PH-triggered controlled ocular drug delivery. Eur. J. Pharm. Biopharm..

[16] Silva D., de Sousa H.C., Gil M.H., Santos L.F., Oom M.S., Alvarez-Lorenzo C. (2021). Moxifloxacin-imprinted silicone-based hydrogels as contact lens materials for extended drug release. Eur. J. Pharmaceut. Sci..

[17] Pereira-da-Mota A.F., Vivero-Lopez M., Topete A., Serro A.P., Concheiro A., Alvarez-Lorenzo C. (2021). Atorvastatin-eluting contact lenses: effects of molecular imprinting and sterilization on drug loading and release. Pharmaceutics.

[18] Maulvi F.A., Shaikh A.A., Lakdawala D.H., Desai A.R., Pandya M.M., Singhania S.S. (2017). Design and optimization of a novel implantation technology in contact lenses for the treatment of dry eye syndrome: in vitro and in vivo evaluation. Acta Biomater..

[19] Rykowska I., Nowak I., Nowak R. (2021). Soft contact lenses as drug delivery systems: a review. Molecules.

[20] Franco P., De Marco I. (2021). Contact lenses as ophthalmic drug delivery systems: a review. Polymers.

[21] Pereira-da-Mota A.F., Vivero-Lopez M., Serramito M., Diaz-Gomez L., Serro A.P., Carracedo G. (2022). Contact lenses for pravastatin delivery to eye segments: design and in vitro-in vivo correlations. J. Contr. Release.

[22] Pereira-da-Mota A.F., Phan C.-M., Concheiro A., Jones L., Alvarez-Lorenzo C. (2022). Testing drug release from medicated contact lenses: the missing link to predict in vivo performance. J. Contr. Release.

[23] Wuchte L.D., DiPasquale S.A., Byrne M.E. (2021). In vivo drug delivery via contact lenses: the current state of the field from origins to present. J. Drug Deliv. Sci. Technol..

[24] Abdi B., Mofidfar M., Hassanpour F., Kirbas Cilingir E., Kalajahi S.K., Milani P.H. (2023). Therapeutic contact lenses for the treatment of corneal and ocular surface diseases: advances in extended and targeted drug delivery. Int. J. Pharm..

[25] Liu Z., Overton M., Chauhan A. (2022). Transport of vitamin E from ethanol/water solution into contact lenses and impact on Drug Transport. J. Ocul. Pharmacol. Therapeut..

[26] Li C.-C., Chauhan A. (2006). Modeling ophthalmic drug delivery by soaked contact lenses. Ind. Eng. Chem. Res..

[27] Torres-Luna C., Fan X., Domszy R., Hu N., Wang N.S., Yang A. (2020). Hydrogel-based ocular drug delivery systems for hydrophobic drugs. Eur. J. Pharmaceut. Sci..

[28] Guzman-Aranguez A., Colligris B., Pintor J. (2013). Contact lenses: promising devices for ocular drug delivery. J. Ocul. Pharmacol. Therapeut..

[29] Holgado M.A., Anguiano-Domínguez A., Martín-Banderas L. (2020).

[30] Aldenhoff Y.B.J., Kruft M.-A.B., Paul Pijpers A., van der Veen F.H., Bulstra S.K., Kuijer R. (2002). Stability of radiopaque iodine-containing biomaterials. Biomaterials.

[31] Hewitt M.G., Morrison P.W., Boostrom H.M., Morgan S.R., Fallon M., Lewis P.N. (2020). In vitro topical delivery of chlorhexidine to the cornea: enhancement using drug-loaded contact lenses and β-cyclodextrin complexation, and the importance of simulating tear irrigation. Mol. Pharm..

[32] Borman P., Elder D. (2017).

[33] Aldenhoff Y.B.J., Kruft M.-A.B., Paul Pijpers A., van der Veen F.H., Bulstra S.K., Kuijer R. (2002). Stability of radiopaque iodine-containing biomaterials. Biomaterials.

[34] Saralidze K., Knetsch M.L., van der Marel C., Koole L.H. (2010). Versatile polymer microspheres for injection therapy: aspects of fluoroscopic traceability and biofunctionalization. Biomacromolecules.

[35] Saralidze K., Knetsch M.L.W., van Berkel R.G.M., Mostert C., Koole L.H. (2011). Radiopaque microspheres for improved transarterial chemical embolization (TACE). J. Contr. Release.

[36] van Hooy-Corstjens C.S., Saralidze K., Knetsch M.L., Emans P.J., de Haan M.W., Magusin P.C. (2007). New intrinsically Radiopaque hydrophilic microspheres for embolization: synthesis and characterization. Biomacromolecules.

[37] Karina A., Benzina A., Tazhibayeva S., Fan H., Koole L.H. (2020). Polymer microparticles with a cavity designed for transarterial chemo‐embolization with crystalline drug formulations. J. Biomed. Mater. Res. B Appl. Biomater..

[38] Morgan S.R., Pilia N., Hewitt M., Moses R.L., Moseley R., Lewis P.N. (2020). Controlled in vitro delivery of voriconazole and diclofenac to the cornea using contact lenses for the treatment of Acanthamoeba keratitis. Int. J. Pharm..

[39] Gokmen M.T., Du Prez F.E. (2012). Porous polymer particles—a comprehensive guide to synthesis, characterization, functionalization and applications. Prog. Polym. Sci..

[40] Van Santvliet L., Ludwig A. (2004). Determinants of eye drop size. Surv. Ophthalmol..

[41] Holland E.J., Darvish M., Nichols K.K., Jones L., Karpecki P.M. (2019). Efficacy of topical ophthalmic drugs in the treatment of dry eye disease: a systematic literature review. Ocul. Surf..

[42] Lai J.-Y., Luo L.-J., Nguyen D.D. (2020). Multifunctional Glutathione-dependent hydrogel eye drops with enhanced drug bioavailability for glaucoma therapy. Chem. Eng. J..

[43] Ponrathnam T., Behere I., Ponrathnam S., Ingavle G. (2023). Highly interconnected porous monolithic and beaded polymers using high internal phase emulsion polymerization: tuning porous architecture through synthesis variables. Polym. Int..

[44] Lanier O.L., Manfre M.G., Bailey C., Liu Z., Sparks Z., Kulkarni S. (2021). Review of approaches for increasing ophthalmic bioavailability for eye drop formulations. AAPS PharmSciTech.

[45] Awwad S., Mohamed Ahmed A.H., Sharma G., Heng J.S., Khaw P.T., Brocchini S. (2017). Principles of pharmacology in the eye. Br. J. Pharmacol..

[46] Shirasaki Y. (2008). Molecular design for enhancement of ocular penetration. J. Pharmaceut. Sci..

[47] Mannermaa E., Vellonen K.-S., Urtti A. (2006). Drug transport in corneal epithelium and blood–retina barrier: emerging role of transporters in ocular pharmacokinetics. Adv. Drug Deliv. Rev..

[48] Mohammadi S., Jones L., Gorbet M. (2014). Extended Latanoprost release from Commercial Contact Lenses: in vitro studies using corneal models. PLoS One.

[49] Maki K.L., Ross D.S. (2014). Exchange of tears under a contact lens is driven by distortions of the contact lens. Integr. Comp. Biol..

[50] Mahomed A., Wolffsohn J.S., Tighe B.J. (2016). Structural design of contact lens-based drug delivery systems; in vitro and in vivo studies of ocular triggering mechanisms. Contact Lens Anterior Eye.

