# Supplementary material for: A novel approach to achieve semi-sustained drug delivery to the eye through asymmetric loading of soft contact lenses
Source: Heliyon. 2023 Jun 5;9(6):e16916. doi: 10.1016/j.heliyon.2023.e16916 (PMC10360931; doi:10.1016/j.heliyon.2023.e16916)
Supplement: Multimedia component 1 [file mmc1.docx]

**Supporting information**


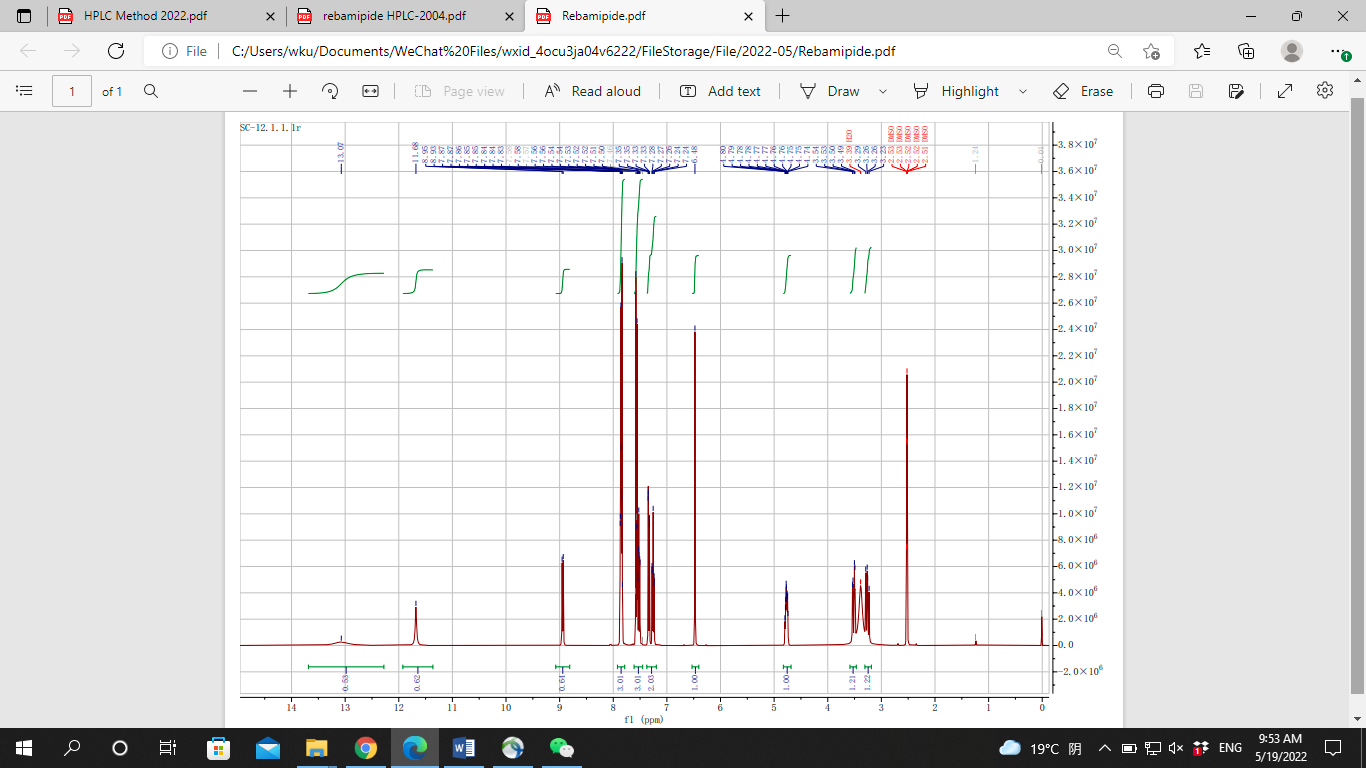


Fig. S1. ^1^H NMR spectra of rebamipide.


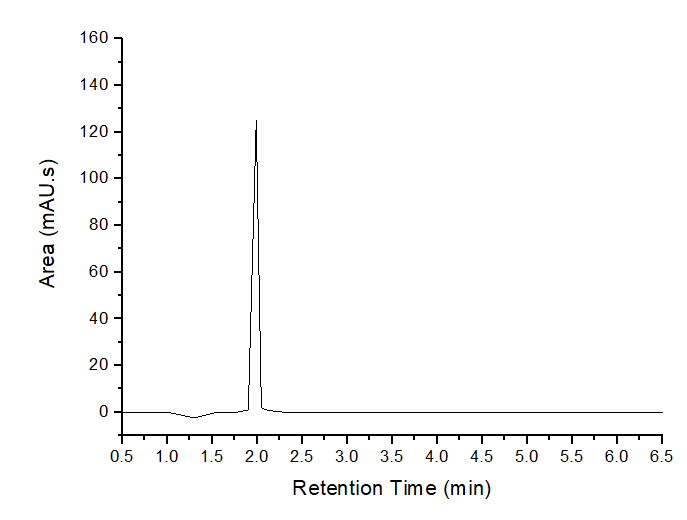


Fig. S2. HPLC-UV analysis results. Chromatogram of rebamipide (Sample concentration 10 µg/mL, retention time 2 min).

Fig. S3. Calibration standards curve of rebamipide made with the following concentrations 1, 5, 10, 25, 50, 75, 100 µg/mL, correlation factor R^2^=0.9999.

*Effects of column temperature and pH*

Effects of column temperature and buffer pH on the peak area and retention time were investigated. Increasing the column temperature (from 35°C to 45°C) led to a shorter retention time (1.62 min) yet unchanged peak areas. On the other hand, modifying the pH of the mobile phase (from pH 4.4 to pH 2.1-5.6) resulted in a broad peak and tailing while the retention time remained unchanged (~2 min). Consequently, the temperature of the column was set at 35°C, and the acetate buffer’s pH was adjusted to pH 4.4 as they showed a good peak resolution.

*Effects of flow rate and composition of the mobile phase*

Flow rate was varied in the range of 0.5–1.5 mL min^−1^. Flow rates of less than 1 mL min^−1^ led to a longer retention time (3.5 min), whereas a flow rate of ≥ 1.2 ml min^−1^ resulted in a lower resolution of the peak. A flow rate of 1 mL min^−1^ was chosen, as it showed the highest theoretical plates within a short retention time (2 min). Increasing the % of acetonitrile (from 30 % to 40-60 % or decreasing the % of methanol from 15 % to 0-10 % resulted in a broad peak and poor resolution.

*Limit of detection (LOD) and limit of quantification (LOQ)*

The LOD and LOQ values were determined with an approach stated by ICH guidelines [36]: the visual evaluation. The LOQ by analyzing samples with pre-known concentrations of analyte and establishing the minimum level at which the analyte can be quantified with acceptable accuracy and precision. The detection limit was defined as the lowest concentration level resulting in a peak area of three times the baseline noise.

*Accuracy and precision*

Three different concentrations of rebamipide were analyzed for quality control (QC) samples; 5 and 10, and 25 μg/mL. Accuracy was expressed as % recovery of rebamipide from QC samples as follows: % accuracy = (C_obs_/C_theor_) ×100, where C_obs_ is the observed concentration of a QC and C_theor_is the theoretical concentration. Precision was defined as the replicates' relative standard deviation (RSD). Intraday precision was calculated by analyzing QC samples in six replicates on the same day, whereas, inter-day precision was assessed on three different days. The obtained %RSD and %Recovery values were less than 2 and between 98-101, respectively, indicating high accuracy, repeatability, and inter day precision of the developed method.

*Linearity*

The linearity between the peak area and concentration was analyzed using a calibration curve obtained from standard solutions of rebamipide (1 to 100 μg/mL). In addition, the correlation coefficient (R^2^) of the regression line with the linear regression equation were determined R^2^ = 0.999; y = 16.73x - 2.504, see Supplementary Material.

*Stability*

To study long-term stability, a solution of rebamipide was prepared and analyzed initially and after 1, 2, and 3 weeks (as per the specified method) by keeping the solution at room temperature. Thus, it was shown that the samples stayed stable for as long as 3 weeks.

*Specificity*

The specificity of the described method was confirmed by the analysis of RBP in the eye drop Mucosta. No interference was observed from the eye drop’s excipients (PVA, sodium citrate dihydrate, KCl, NaCl, HCl) which shows a high specificity of the method.


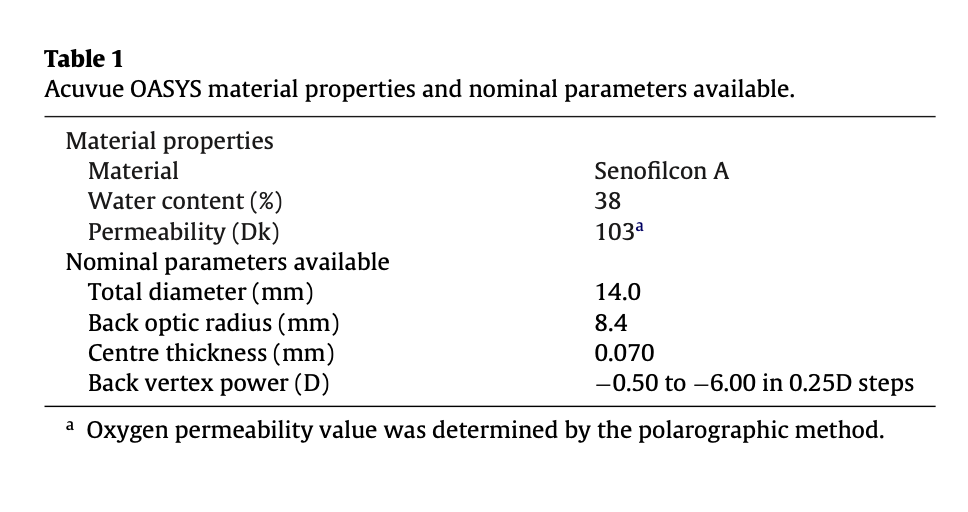
Fig. S4. Acuvue Oasys material properties and nominal parameters. Available From: M. Guillon, C. Maïssa, Long term effects of the daily wear of senofilcon a silicone hydrogel contact lenses on eyelid tissues, Contact Lens and Anterior Eye. 35 (2012) 112–117.
